# Supplementary material for: Pyruvate Dehydrogenase Kinase 1 inhibition mediated oxidative phosphorylation enhancement in cartilage promotes osteoarthritis progression
Source: BMC Musculoskelet Disord. 2023 Jul 20;24:597. doi: 10.1186/s12891-023-06585-6 (PMC10357736; doi:10.1186/s12891-023-06585-6)
Supplement: Supplementary file 1 — Additional file 1. [file 12891_2023_6585_MOESM1_ESM.zip › KEGG.Gsea.1653622432464/KEGG_GLUTATHIONE_METABOLISM.html]

Details for gene set KEGG\_GLUTATHIONE\_METABOLISM[GSEA]

|  || Dataset | 55457\_collapsed\_to\_symbols.cls.cls #OA\_versus\_normal.cls.cls #OA\_versus\_normal\_repos |
| Phenotype | cls.cls#OA\_versus\_normal\_repos |
| Upregulated in class | OA |
| GeneSet | KEGG\_GLUTATHIONE\_METABOLISM |
| Enrichment Score (ES) | 0.3623822 |
| Normalized Enrichment Score (NES) | 1.3701913 |
| Nominal p-value | 0.07524272 |
| FDR q-value | 0.12936144 |
| FWER p-Value | 0.978 |
Table: GSEA Results Summary

  

Fig 1: Enrichment plot: KEGG\_GLUTATHIONE\_METABOLISM      
 Profile of the Running ES Score & Positions of GeneSet Members on the Rank Ordered List

  

| SYMBOL | TITLE | RANK IN GENE LIST | RANK METRIC SCORE | RUNNING ES | CORE ENRICHMENT || 1 | GSTA4 | glutathione S-transferase alpha 4 [Source:HGNC Symbol;Acc:HGNC:4629] | 216 | 0.808 | 0.0537 | Yes |
| 2 | GSTK1 | glutathione S-transferase kappa 1 [Source:HGNC Symbol;Acc:HGNC:16906] | 490 | 0.662 | 0.0903 | Yes |
| 3 | GSS | glutathione synthetase [Source:HGNC Symbol;Acc:HGNC:4624] | 502 | 0.658 | 0.1470 | Yes |
| 4 | MGST3 | microsomal glutathione S-transferase 3 [Source:HGNC Symbol;Acc:HGNC:7064] | 610 | 0.620 | 0.1928 | Yes |
| 5 | IDH2 | isocitrate dehydrogenase (NADP(+)) 2 [Source:HGNC Symbol;Acc:HGNC:5383] | 967 | 0.525 | 0.2110 | Yes |
| 6 | GSTZ1 | glutathione S-transferase zeta 1 [Source:HGNC Symbol;Acc:HGNC:4643] | 974 | 0.523 | 0.2562 | Yes |
| 7 | GSTM3 | glutathione S-transferase mu 3 [Source:HGNC Symbol;Acc:HGNC:4635] | 1025 | 0.512 | 0.2970 | Yes |
| 8 | GSTT1 | glutathione S-transferase theta 1 [Source:HGNC Symbol;Acc:HGNC:4641] | 1129 | 0.491 | 0.3319 | Yes |
| 9 | GSTP1 | glutathione S-transferase pi 1 [Source:HGNC Symbol;Acc:HGNC:4638] | 1260 | 0.465 | 0.3624 | Yes |
| 10 | GPX7 | glutathione peroxidase 7 [Source:HGNC Symbol;Acc:HGNC:4559] | 1919 | 0.370 | 0.3435 | No |
| 11 | MGST2 | microsomal glutathione S-transferase 2 [Source:HGNC Symbol;Acc:HGNC:7063] | 2583 | 0.292 | 0.3175 | No |
| 12 | LAP3 | leucine aminopeptidase 3 [Source:HGNC Symbol;Acc:HGNC:18449] | 3044 | 0.245 | 0.3032 | No |
| 13 | GSTM5 | glutathione S-transferase mu 5 [Source:HGNC Symbol;Acc:HGNC:4637] | 3123 | 0.238 | 0.3179 | No |
| 14 | GSTM4 | glutathione S-transferase mu 4 [Source:HGNC Symbol;Acc:HGNC:4636] | 3144 | 0.235 | 0.3368 | No |
| 15 | RRM2 | ribonucleotide reductase regulatory subunit M2 [Source:HGNC Symbol;Acc:HGNC:10452] | 3505 | 0.202 | 0.3265 | No |
| 16 | GPX5 | glutathione peroxidase 5 [Source:HGNC Symbol;Acc:HGNC:4557] | 3558 | 0.198 | 0.3397 | No |
| 17 | GSTA3 | glutathione S-transferase alpha 3 [Source:HGNC Symbol;Acc:HGNC:4628] | 3807 | 0.176 | 0.3358 | No |
| 18 | RRM1 | ribonucleotide reductase catalytic subunit M1 [Source:HGNC Symbol;Acc:HGNC:10451] | 4501 | 0.113 | 0.2917 | No |
| 19 | GSR | glutathione-disulfide reductase [Source:HGNC Symbol;Acc:HGNC:4623] | 4597 | 0.105 | 0.2935 | No |
| 20 | GGT1 | gamma-glutamyltransferase 1 [Source:HGNC Symbol;Acc:HGNC:4250] | 5177 | 0.058 | 0.2536 | No |
| 21 | OPLAH | "5-oxoprolinase, ATP-hydrolysing [Source:HGNC Symbol;Acc:HGNC:8149]" | 5339 | 0.045 | 0.2449 | No |
| 22 | GPX2 | glutathione peroxidase 2 [Source:HGNC Symbol;Acc:HGNC:4554] | 5808 | 0.008 | 0.2092 | No |
| 23 | GSTM1 | glutathione S-transferase mu 1 [Source:HGNC Symbol;Acc:HGNC:4632] | 5848 | 0.005 | 0.2067 | No |
| 24 | ODC1 | ornithine decarboxylase 1 [Source:HGNC Symbol;Acc:HGNC:8109] | 6153 | -0.016 | 0.1845 | No |
| 25 | GSTO1 | glutathione S-transferase omega 1 [Source:HGNC Symbol;Acc:HGNC:13312] | 6275 | -0.025 | 0.1773 | No |
| 26 | G6PD | glucose-6-phosphate dehydrogenase [Source:HGNC Symbol;Acc:HGNC:4057] | 7082 | -0.086 | 0.1221 | No |
| 27 | IDH1 | isocitrate dehydrogenase (NADP(+)) 1 [Source:HGNC Symbol;Acc:HGNC:5382] | 7109 | -0.089 | 0.1279 | No |
| 28 | GPX4 | glutathione peroxidase 4 [Source:HGNC Symbol;Acc:HGNC:4556] | 7115 | -0.089 | 0.1353 | No |
| 29 | GSTT2 | glutathione S-transferase theta 2 (gene/pseudogene) [Source:HGNC Symbol;Acc:HGNC:4642] | 7666 | -0.132 | 0.1040 | No |
| 30 | SRM | spermidine synthase [Source:HGNC Symbol;Acc:HGNC:11296] | 7735 | -0.136 | 0.1106 | No |
| 31 | GCLC | glutamate-cysteine ligase catalytic subunit [Source:HGNC Symbol;Acc:HGNC:4311] | 8534 | -0.196 | 0.0657 | No |
| 32 | SMS | spermine synthase [Source:HGNC Symbol;Acc:HGNC:11123] | 8884 | -0.223 | 0.0581 | No |
| 33 | GGT5 | gamma-glutamyltransferase 5 [Source:HGNC Symbol;Acc:HGNC:4260] | 9361 | -0.261 | 0.0439 | No |
| 34 | GCLM | glutamate-cysteine ligase modifier subunit [Source:HGNC Symbol;Acc:HGNC:4312] | 10439 | -0.366 | -0.0080 | No |
| 35 | GGCT | gamma-glutamylcyclotransferase [Source:HGNC Symbol;Acc:HGNC:21705] | 10515 | -0.375 | 0.0190 | No |
| 36 | PGD | phosphogluconate dehydrogenase [Source:HGNC Symbol;Acc:HGNC:8891] | 10845 | -0.410 | 0.0292 | No |
| 37 | ANPEP | "alanyl aminopeptidase, membrane [Source:HGNC Symbol;Acc:HGNC:500]" | 11404 | -0.487 | 0.0284 | No |
| 38 | GPX3 | glutathione peroxidase 3 [Source:HGNC Symbol;Acc:HGNC:4555] | 11407 | -0.488 | 0.0709 | No |
| 39 | GSTA1 | glutathione S-transferase alpha 1 [Source:HGNC Symbol;Acc:HGNC:4626] | 11608 | -0.517 | 0.1005 | No |
Table: GSEA details [plain text format]

  

Fig 2: KEGG\_GLUTATHIONE\_METABOLISM      
 Blue-Pink O' Gram in the Space of the Analyzed GeneSet

  

Fig 3: KEGG\_GLUTATHIONE\_METABOLISM: Random ES distribution      
 Gene set null distribution of ES for **KEGG\_GLUTATHIONE\_METABOLISM**
